# Diet–Gene Interaction Between Fruit Intake and *CMIP* rs2925979 Polymorphism in Relation to Type 2 Diabetes: A Family-Based Study in Northern China

**DOI:** 10.3390/nu17111789

**Published:** 2025-05-24

**Authors:** Liangchun Kuo, Yinxi Tan, Yiqun Wu, Xueying Qin, Haiying Gong, Yao Zhao, Tao Wu, Dafang Chen, Mengying Wang, Junbo Wang, Yonghua Hu

**Affiliations:** 1 Department of Nutrition and Food Hygiene, School of Public Health, Peking University, Beijing 100191, China; kuo_liangchun@bjmu.edu.cn (L.K.); tanyinxi@bjmu.edu.cn (Y.T.); 2 Department of Epidemiology and Biostatistics, School of Public Health, Peking University, Beijing 100191, China; qywu118@163.com (Y.W.); xueyingqin@gmail.com (X.Q.); twu@bjmu.edu.cn (T.W.); dafangchen@bjmu.edu.cn (D.C.); yhhu@bjmu.edu.cn (Y.H.); 3 Key Laboratory of Epidemiology of Major Diseases, Peking University, Ministry of Education, Beijing 100191, China; 4 Beijing Fangshan District Centre for Disease Control and Prevention, Beijing 102446, China; gonghaiying2802862@163.com (H.G.); yue1112@163.com (Y.Z.); 5 Beijing Key Laboratory of Toxicological Research and Risk Assessment for Food Safety, Peking University, Beijing 100191, China

**Keywords:** fruit intake, type 2 diabetes, *CMIP*, gene-diet interactions

## Abstract

**Background/Objectives**: This study aimed to investigate the association between dietary intake and the risk of type 2 diabetes mellitus (T2DM) in a rural northern Chinese population, and to explore potential gene–diet interactions that may influence T2DM susceptibility. **Methods**: A total of 1747 participants (1138 with T2DM and 609 without) were included, using baseline data from a family-based cohort study in rural northern China. Demographic characteristics, lifestyle factors, and medical history were collected via standardized questionnaires. Dietary intake was assessed using a semi-quantitative food frequency questionnaire, and anthropometric measurements were conducted according to standardized protocols. Based on findings from previous genome-wide association studies, several T2DM-related single-nucleotide polymorphisms were selected for genotyping. Generalized linear models accounting for familial clustering were employed to examine the associations between dietary intake and T2DM risk, and to assess gene–diet interaction. **Results**: A significant inverse association was observed between fruit intake and T2DM risk. Furthermore, a significant interaction was found between fruit consumption and the *CMIP* rs2925979 polymorphism: the protective effect of higher fruit intake was evident among individuals carrying the T allele but not among those with the CC genotype. **Conclusions**: These findings suggest that genetic variation may modify metabolic responses to dietary factors, particularly fruit intake. The results underscore the importance of considering gene–diet interactions in the prevention of T2DM.

## 1. Introduction

Type 2 diabetes mellitus (T2DM) accounts for over 90% of all diabetes cases globally, and is the most prevalent and fastest-growing form of the disease. Diagnosis of T2DM is based on criteria established by the American Diabetes Association (ADA) [1] and the World Health Organization (WHO), which include fasting plasma glucose ≥ 7.0 mmol/L, 2-h plasma glucose ≥ 11.1 mmol/L after an oral glucose tolerance test (OGTT), or glycated hemoglobin (HbA1c) ≥ 6.5% [2].

According to the International Diabetes Federation (IDF), an estimated 537 million people worldwide are currently living with diabetes. This number is projected to rise to 643 million by 2030 and 783 million by 2045 [1]. In China, the number of people with diabetes increased from 90 million in 2011 to 118 million in 2021, representing a 56% increase [3,4,5,6]. Over the past three decades, five nationally representative cross-sectional studies have shown a steady upward trend in diabetes prevalence. In 1980, the prevalence of diabetes in China was less than 1%; by 2010, it had risen to 11.6%, surpassing the 10% threshold and placing China among the countries with the highest diabetes burden [3,4,5,6]. This persistent rise underscores the urgency of identifying modifiable and non-modifiable risk factors contributing to the growing T2DM epidemic [7,8,9,10].

In recent years, genome-wide association studies (GWAS) have identified more than 400 genetic loci associated with T2DM risk. One gene of particular interest is the c-Maf inducing protein (*CMIP*) gene, which plays a role in T-cell signaling, lipid metabolism, and insulin resistance pathways [11,12]. The single-nucleotide polymorphism (SNP) rs2925979, located in an intronic region of the *CMIP* gene [11], has been identified as a susceptibility locus for T2DM in East Asian populations. Notably, studies have shown that although the T allele of rs2925979 is inversely associated with body mass index (BMI) and waist circumference, it is positively associated with T2DM risk in Chinese women, suggesting pleiotropic and sex-specific effects [13]. Moreover, rs2925979 has been linked to lipid metabolism and adiponectin levels, indicating a broader role in metabolic regulation [14,15,16].

Dietary factors, particularly fruit consumption, are well-established in the prevention of T2DM. Fruits are rich in dietary fiber, polyphenols, and antioxidants, which collectively improve insulin sensitivity and reduce systemic inflammation [17]. Large-scale cohort studies and meta-analyses have consistently demonstrated an inverse relationship between fruit intake and T2DM risk [18,19,20]. However, interindividual variability in metabolic responses to dietary factors suggests a possible gene–diet interaction. To date, few studies have examined the interaction between *CMIP* rs2925979 and fruit intake in relation to T2DM risk, particularly in nutritionally and genetically distinct populations such as rural Chinese residents.

This study examined the potential modifying effect of the *CMIP* rs2925979 polymorphism on the association between fruit intake and the risk of T2DM, using data from a large family-based cohort study conducted in rural northern China. Given the increasing burden of T2DM in rural populations with limited healthcare access, and the paucity of research on how genetic variation may influence dietary associations in these settings, this investigation addresses a critical knowledge gap by evaluating the interaction between genetic susceptibility and modifiable dietary factors. Exploring gene–diet interactions is essential for advancing precision nutrition and informing population-specific preventive strategies.

We hypothesize that higher fruit intake is associated with a lower risk of T2DM, and that this protective association is more pronounced among carriers of the T allele of the *CMIP* rs2925979 polymorphism, compared to individuals with the CC (homozygous major allele) genotype. This study investigates the independent and interactive associations of fruit intake and the *CMIP* rs2925979 polymorphism with T2DM risk, aiming to generate evidence to inform personalized dietary recommendations and targeted prevention strategies in rural Chinese populations.

## 2. Materials and Methods

### 2.1. Study Participants

This study used baseline data from the Family Cohort Study of Common Chronic Non-Communicable Diseases in Rural Northern China, as previously described. Nine administrative villages in Fangshan District, Beijing, were selected as study sites due to their demographic, economic, and geographical characteristics representative of northern China. From June 2005 to August 2017, a total of 8323 participants were recruited using a family-based recruitment approach. All participants completed standardized questionnaires, underwent physical measurements, and provided blood samples. The cohort was designed based on genetic relationships, facilitating a more effective assessment of environmental and lifestyle factors while controlling for genetic background differences. The inclusion criteria for this study were: (1) age ≥ 18 years at the time of enrollment; (2) complete dietary survey data; (3) genetic testing data; and (4) voluntary participation with the completion of questionnaires, physical examinations, and biochemical tests. Exclusion criteria included: severe diseases or chronic conditions, such as malignant tumors or severe liver or kidney diseases, that hinder study participation. After applying the inclusion and exclusion criteria, 1747 participants were enrolled, fulfilling the sample size requirement. All participants provided informed consent, and the study was approved by the Biomedical Ethics Committee of Peking University (Approval No: IRB00001052-13027).

### 2.2. Dietary Assessment

The study employed a semi-quantitative food frequency questionnaire (FFQ) adapted from a validated simplified Chinese version developed for Chinese populations, and further modified to reflect the dietary characteristics of the study population. The questionnaire covered intake of key food groups, including staple foods (e.g., rice, noodles), vegetables, fruits, animal-based foods (e.g., unprocessed meat, processed meat, seafood, eggs), plant-based protein sources (e.g., tofu, nuts), and dairy products. It assessed both the frequency (e.g., days per week) and portion size (e.g., grams per day) of food consumption over the past 12 months. Additional questions addressed beverage intake (alcohol, tea, sugar-sweetened beverages), cooking oil types, salt consumption, and household eating patterns, enabling a comprehensive characterization of dietary behaviors relevant to chronic disease risk.

### 2.3. Genotyping and Basic Information on Polymorphic Loci

Based on previous GWAS conducted in European and East Asian populations, this study screened genetic susceptibility loci that reached genome-wide significance (*p* < 5 × 10^−8^) for associations with T2DM and its major metabolic risk factors, including blood pressure, blood lipid levels [11,21,22], insulin resistance [23,24,25], and obesity [26,27,28,29]. Inclusion criteria for SNP selection were as follows: (1) SNPs identified with genome-wide significance (*p* < 5 × 10^−8^) in GWAS for T2DM or its associated metabolic traits; (2) SNPs discovered in East Asian populations were prioritized to enhance population relevance, although variants from European GWAS were included due to the limited availability of East Asian-specific findings; and (3) SNPs previously associated with increased risk of T2DM (i.e., odds ratio [OR] or relative risk [RR] > 1), to facilitate risk-oriented analysis. Exclusion criteria included: (1) SNPs lacking replication in independent GWAS or validation cohorts; (2) SNPs with genotyping call rates below 95% or with >5% missing data; and (3) SNPs exhibiting high linkage disequilibrium (r^2^ > 0.8) with other selected loci, to reduce redundancy. After applying these criteria, SNP rs2925979 in the *CMIP* gene was selected for genotyping, based on its previously reported association with T2DM risk in East Asian populations. The distribution of *CMIP* rs2925979 genotypes was assessed for Hardy–Weinberg Equilibrium (HWE) using a chi-square test, and no significant deviation was detected (*p* = 0.514), suggesting that the genotype distribution was representative and free from genotyping bias.

### 2.4. Definition and Standards of Relevant Indicators

The covariate information for this study was obtained from a questionnaire, which included variables such as age, gender, marital status, education level, occupation, waist circumference, hip circumference, blood pressure, stroke history, hypertension history, BMI, smoking status, drinking status, and regular exercise. Waist and hip circumferences were measured with a precision of 0.1 cm. Blood pressure was measured three times with a 1-min interval between measurements, with the first measurement discarded and the average of the last two measurements used as the final value. All measurements were performed by the same researcher using the same blood pressure monitor. Stroke and hypertension histories were recorded as binary variables (‘yes’ or ‘no’). BMI was calculated from height and weight, with height measured to the nearest 0.1 cm and weight to the nearest 0.1 kg. Smoking status was categorized as “never smoked” or “smoked”, drinking status as “never drank” or “drank”, and regular exercise as ‘yes’ or ‘no’.

### 2.5. Statistical Analysis

Descriptive analyses were conducted on the study participants’ general demographic characteristics, physical measurements, lifestyle behaviors, and personal medical history. For continuous variables following a normal distribution, data were presented as means ± standard deviations; for non-normally distributed variables, medians and interquartile ranges (P_25_, P_75_) were used. Categorical variables were described as frequencies (percentages). Since the study participants were not statistically independent, intergroup comparisons were performed using generalized linear models that accounted for familial clustering. To control for potential confounding factors, a generalized linear regression model was constructed with T2DM status as the dependent variable (coded as 0 = no, 1 = yes) and various food categories as independent variables. The model was adjusted for age, sex, family structure, marital status, educational level, occupation, smoking status, alcohol consumption, regular physical activity, hypertension, BMI, waist circumference, total cholesterol, and stroke history. Interaction terms between food categories and genetic loci were included in the model to evaluate the presence of interaction effects, as indicated by the statistical significance of the interaction terms (*p*-values). Stratified analyses were subsequently conducted according to genotype to assess the associations between individual food categories and T2DM, and to further evaluate the potential effect modification by gene polymorphisms on these associations. All statistical analyses were conducted using R software (version 4.2.2).

## 3. Results

### 3.1. Basic Characteristics

Table 1 shows that 1747 participants were included in the study, comprising 1138 individuals diagnosed with T2DM and 609 without T2DM. Compared to those without T2DM, individuals in the T2DM group were significantly older and exhibited higher mean values for waist circumference, fasting blood glucose, and HbA1c levels. Furthermore, the prevalence of hypertension was significantly greater in the T2DM group (*p* < 0.05). In contrast, the proportions of participants who reported alcohol consumption and regular physical activity were significantly lower among individuals with T2DM (*p* < 0.05). No significant differences were observed between the two groups in terms of sex, occupational status, educational attainment, marital status, hip circumference, BMI, systolic blood pressure, total cholesterol, history of stroke, or smoking status (*p* > 0.05). In terms of genetic distribution, the genotype frequencies of *CMIP* rs2925979 among all participants were as follows: CC, 32.81%; CT, 48.21%; and TT, 18.98%. The genotype distribution used Hardy–Weinberg Equilibrium (*p* = 0.514), supporting the validity of the genotyping results.

### 3.2. Association Between Dietary Intake and T2DM

The intake of each food group was categorized into quartiles (Q1–Q4) based on the amount taken (in grams per day), with the lowest quartile (Q1) serving as the reference group. To evaluate the relationship between dietary intake and the risk of T2DM, three generalized linear models were applied. Model 1 was adjusted for age and sex; Model 2 included additional adjustments for marital status, educational level, and occupation; and Model 3 further accounted for lifestyle and clinical variables, including smoking status, alcohol consumption, regular physical activity, hypertension, BMI, waist circumference, total cholesterol, and history of stroke. As presented in Table 2, fruit intake was inversely associated with the risk of T2DM across all adjusted models. In the fully adjusted Model 3, compared to the reference group (Q1), the odds ratios (ORs) for T2DM were 0.44 (95% CI: 0.33–0.60) for Q2, 0.46 (95% CI: 0.33–0.62) for Q3, and 0.58 (95% CI: 0.43–0.80) for Q4, all of which were statistically significant (*p* < 0.001).

### 3.3. Interaction Between Dietary Factors and Genetic Polymorphisms

**Figure 1:**
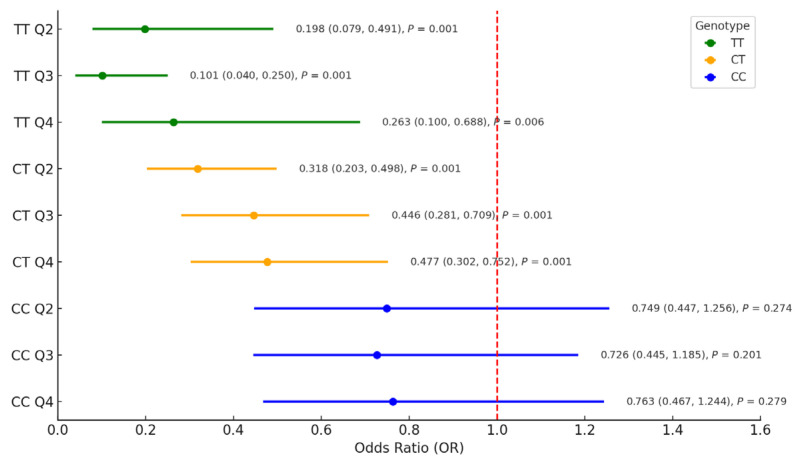
Forest plot of the association between quartiles under fruit intake score and type 2 diabetes risk stratified by *CMIP* gene rs2925979 genotype.

As shown in Figure 1, the association between fruit intake and T2DM risk differed according to *CMIP* rs2925979 genotype (TT, CT, and CC). Among individuals with the TT genotype, higher fruit intake was significantly associated with a reduced risk of T2DM. Compared to the lowest quartile (Q1), the adjusted odds ratios (ORs, 95% confidence intervals [CIs]) for Q2, Q3, and Q4 were 0.198 (0.079–0.491), 0.101 (0.040–0.250), and 0.263 (0.100–0.688), respectively (all *p* < 0.01), indicating a precise inverse dose–response trend. A similar protective pattern was observed among individuals with the CT genotype, with ORs (95% CIs) of 0.318 (0.203–0.498), 0.446 (0.281–0.709), and 0.477 (0.302–0.752) for Q2–Q4 versus Q1, respectively (all *p* < 0.001). In contrast, no significant associations were found among individuals with the CC genotype, with ORs of 0.749, 0.726, and 0.763 for Q2–Q4 (all *p* > 0.05; 95% CIs included 1.0).

Importantly, we also evaluated the independent association between *CMIP* rs2925979 and T2DM risk, irrespective under fruit intake. Compared to the CC genotype, carriers of the T allele (CT or TT) did not show a statistically significant association with T2DM in fully adjusted models (results available upon request), suggesting that the T allele alone may not confer increased risk. These findings indicate a potential gene–diet interaction, in which the T allele’s influence on T2DM risk appears to depend on fruit intake levels.

## 4. Discussion

This study identified a significant inverse association between fruit intake and the risk of T2DM in a rural northern Chinese population. Notably, a significant interaction was observed between fruit intake and the *CMIP* rs2925979 polymorphism. Specifically, the protective effect of higher fruit consumption was evident among individuals carrying the T allele (TT and CT genotypes). In contrast, no significant association was found among those with the CC genotype. These findings suggest a potential gene–diet interaction contributing to interindividual variability in T2DM susceptibility. The observed protective role under fruit intake is consistent with results from large-scale prospective cohort studies and meta-analyses, which have consistently demonstrated an inverse relationship between fruit consumption and T2DM risk [30,31]. Mechanistically, fruits are rich in dietary fiber, polyphenols, and essential micronutrients, all of which have been shown to enhance insulin sensitivity, reduce oxidative stress, and modulate inflammatory pathways [32,33,34]. Furthermore, the inverse association in our study remained robust even after adjusting for a comprehensive set of sociodemographic, lifestyle, and metabolic confounders, supporting an independent protective effect under fruit intake [13,35,36].

This study makes a novel contribution by identifying a significant interaction between fruit intake and the *CMIP* rs2925979 genotype in relation toT2DM risk. *CMIP* (c-Maf-inducing protein) is known to participate in insulin signaling and T-cell activation pathways, and its polymorphisms have been previously associated with insulin resistance and lipid metabolism abnormalities [31,37,38]. Our findings suggest that the stronger inverse association between fruit consumption and T2DM observed among individuals carrying the T allele may reflect differences in gene expression or metabolic responsiveness to dietary bioactive compounds such as flavonoids and dietary fiber. These results are consistent with a growing body of evidence indicating that genetic background can influence metabolic responses to diet, thereby supporting the rationale for precision nutrition strategies [39,40]. Notably, no significant association was found between fruit intake and T2DM risk among individuals with the CC genotype, suggesting that the protective effect of fruit consumption may be diminished by specific genetic factors [16,41,42,43]. These findings underscore the importance of integrating gene–environment interactions into public health nutrition strategies and tailoring dietary recommendations to accommodate genetic variability within populations.

The strengths of this study lie in its use of a family-based cohort design, which effectively reduces population stratification bias, and the comprehensive adjustment for a wide range of potential confounders. Moreover, conducting genotype-stratified analyses enabled the identification of significant gene–diet interactions that might be obscured in analyses of the general population. Despite these strengths, several limitations warrant consideration. First, the cross-sectional nature of the study precludes causal inference. Second, dietary intake was assessed using a food frequency questionnaire (FFQ), which is prone to recall bias and measurement error. Third, the small sample size within the CC genotype subgroup may have limited the statistical power to detect modest associations. Finally, our analysis focused on a single SNP and one food group; thus, future studies should explore genome-wide interactions and incorporate a broader range of dietary exposures to enhance generalizability.

Overall, this study demonstrates a significant gene–diet interaction between fruit intake and the *CMIP* rs2925979 polymorphism in relation to T2DM risk. These findings underscore the need to consider genetic background in formulating dietary recommendations and contribute valuable evidence toward the development of precision nutrition strategies for diabetes prevention.

## 5. Conclusions

In summary, this study presents novel evidence of a gene–diet interaction between fruit intake and the *CMIP* rs2925979 polymorphism in relation to the risk of T2DM in a rural northern Chinese population. The findings highlight the protective effect of fruit consumption and suggest that genetic variations may modify individual susceptibility to T2DM. By emphasizing differential metabolic responses to dietary components based on genetic background, our results strengthen the case for advancing precision nutrition strategies tailored to genetic profiles. These findings provide a scientific basis for more personalized dietary recommendations and contribute to the growing body of research supporting the integration of nutrigenetics into public health strategies aimed at diabetes prevention.

## Figures and Tables

**Table 1:** Demographic Characteristics of Study Subjects [*n* (%)].

Basic Characteristics	Total Number (*n* = 1747)	T2DM (*n* = 1138)	Non-T2DM (*n* = 609)	*p* Value
Age, years, mean (SD)	58.7 ± 8.6	59.1 ± 8.5	57.9 ± 8.8	0.006
Gender, *n* (%)				0.996
Male	677 (38.8)	441 (38.8)	236 (38.8)	
Female	1070 (61.2)	697 (61.2)	373 (61.2)	
Occupation, *n* (%)				0.306
Farmer	538 (30.8)	341 (30.0)	197 (32.3)	
Non-Farmer	1209 (69.2)	797 (70.0)	412 (67.7)	
Education level, *n* (%)				0.654
Junior high school and below	1465 (83.9)	951 (83.6)	514 (84.4)	
High school and above	282 (16.1)	187 (16.4)	95 (15.6)	
Marital status, *n* (%)				0.607
Married	1522 (87.1)	988 (86.8)	534 (87.7)	
Unmarried	225 (12.9)	150 (13.2)	75 (12.3)	
Waist circumference (cm), mean (SD)	92.5 ± 9.4	93.4 ± 9.4	91.0 ± 9.3	<0.001
Hip circumference (cm), mean (SD)	99.7 ± 7.2	99.8 ± 7.4	99.6 ± 6.8	0.675
BMI (kg/m^2^), *n* (%)				0.534
<18.5	25 (1.4)	15 (1.3)	10 (1.6)	
18.50~23.99	446 (25.5)	281 (24.7)	165 (27.1)	
24.00~27.99	767 (43.9)	506 (44.5)	261 (42.9)	
≥28.00	509 (29.1)	336 (29.5)	173 (28.4)	
Systolic blood pressure (mmHg), mean (SD)	135.4 ± 19.0	135.7 ± 18.7	134.9 ± 19.6	0.356
Diastolic blood pressure (mmHg), mean (SD)	80.0 ± 11.2	79.4 ± 11.1	81.2 ± 11.3	0.001
Fasting blood glucose (mmol/L), mean (SD)	6.0 ± 2.9	6.7 ± 3.1	4.7 ± 2.1	<0.001
Total cholesterol (mmol/L), mean (SD)	2.8 ± 1.2	2.8 ± 1.2	2.9 ± 1.1	0.154
HbA1c (%, mean (SD))	7.2 ± 1.8	7.8 ± 1.7	6.0 ± 1.1	<0.001
Stroke history, *n* (%)				0.202
Yes	297 (17.0)	184 (16.2)	113 (18.6)	
No	1450 (83.0)	954 (83.3)	496 (81.4)	
Hypertension, *n* (%)				0.002
Yes	1222 (69.9)	824 (72.4)	398 (65.4)	
No	525 (30.1)	314 (27.6)	211 (34.6)	
Smoking, *n* (%)				0.872
Never smoked	1097 (62.8)	713 (62.7)	384 (63.1)	
Smoking	650 (37.2)	425 (37.3)	225 (36.9)	
Drinking, *n* (%)				0.015
Never drank	1148 (65.7)	771 (67.8)	377 (61.9)	
Drank	599 (34.3)	367 (32.2)	232 (38.1)	
Regular exercise, *n* (%)				0.015
Yes	308 (17.6)	182 (16.0)	126 (20.7)	
No	1439 (82.4)	956 (84.0)	483 (79.3)	

Continuous variables are presented as mean ± standard deviation, categorical variables as frequency (percentage). *p*-values were calculated using *t*-tests (for continuous variables) or chi-square tests (for categorical variables).

**Table 2:** The Association Between Fruit Intake and the Risk of T2DM.

Intake Level	Model 1		Model 2		Model 3	
	OR (95% CI)	*p* Value	OR (95% CI)	*p* Value	OR (95% CI)	*p* Value
Q1	1.00		1.00		1.00	
Q2	0.45 (0.34, 0.61)	<0.001	0.45 (0.34, 0.61)	<0.001	0.44 (0.33, 0.60)	<0.001
Q3	0.47 (0.35, 0.62)	<0.001	0.46 (0.40, 0.61)	<0.001	0.46 (0.33, 0.62)	<0.001
Q4	0.60 (0.44, 0.81)	<0.001	0.59 (0.43, 0.79)	<0.001	0.58 (0.43, 0.80)	<0.001

## Data Availability

The data presented in this study are available on request from the corresponding author. The data are not publicly available due to privacy.
